# Twelfth Annual Session of the American Homœopathic Institute, Held at Buffalo, June 7th and 8th

**Published:** 1855-07

**Authors:** 


*Twelfth Annual Session of the American Homœopathic Institute, held at Buffalo, June 7th and 8th.*—During the early days of June, the daily papers of our goodly town gave note of preparation of this momentous occasion. Hotel keepers had heard of the rush of M. D.'s at Philadelphia, in May, and laid in extra roasts of beef. Reporters for the daily press were all agog. Especially important were the faces of the half dozen resident homœopathic practitioners. When we saw the anxious cares that sate upon their brows, we were moved to sympathy.

The day arrived, as all days do except that of the Second Advent. About 10, A. M., two ladies, escorted by a pot-bellied philosopher with very roomy breeches and a huge cane, made a solemn entry into American Hall, which contained seats for 1200 persons. The crowd gradually increased until nineteen gentlemen were in attendance, when the roll was called, six of whom had the honor of representing Buffalo. The *tout-ensemble* at this moment was imposing. A disciple of Lavater present, noticed a physiognomonical peculiarity in the noses of members. The nasal organ of each and every delegate was of the elongated Hebrew style, indicating a cross between the live Yankee and the Chatham St. "old clo' " man.

After the calling of the roll most of the members of the Institute were elected officers for the ensuing year. The Treasurer made a report, and no defalcations being evident, the Institute adjourned to 3, P. M.

*Afternoon Session.* Two or three gentlemen read reports said to be very interesting. (Singular fact that reporters for the press are very easily interested.) Twelve new members were reported, including one addition to the already numerous and talented delegation from Buffalo. The next business in order was reports of committees. No committees reported except Dr. F. Humphreys from the "Bureau on the Augmentation and Improvement of the Materia Medica." ("Big, fat words," were in order at this meeting.)

Then a resolution was introduced intimating that Dr. Humphreys himself had been augmenting the materia medica in the way of advertising and vending certain homœopathic nostrums, called the "New Era Medicines," and a committee was appointed to post up the Institute on the subject.

The Institute then adjourned to 7½, P. M., to listen to the address of the President. At 8, P. M., we repaired to the Hall. Three ladies and a couple of dozen gentlemen where in attendance. When we entered, they left; and the gas being immediately turned off, we were left in the dark as to the order of proceedings.

June 8. Morning papers announce that the annual address is to be delivered *this* evening—"no preventing Providence," of course, understood.

*Morning Session.* Dr. F. Humphreys is on the anxious seat, accused of unprofessional conduct. Current of opinion runs against the doctor.

*Afternoon Session.* Dr. Humphreys refuses to make any concession, and leaves the room breathing threatenings and slaughter. His outraged feelings find "surcease of sorrow," however, in the prospect of sundry fat libel suits against the Institute, or its members. Libel suits are among the most humane of civilized institutions, and seem especially provided for the consolation of afflicted scamps whose sins have found them out.

Dr. Humphreys having left, the Institute were so unkind as to expel him formally. After this, the great incident in the annals of this meeting, the

Institute passed a resolution or two of thanks, and then adjourned. The annual address is still waiting delivery for the want of an audience.

And so terminated the most feeble attempt at respectability in point of numbers, appearance, talent, or enthusiasm, we have ever witnessed. It was dismal and cheerless beyond description.

